# Lactylation in cancer: metabolic mechanism and therapeutic strategies

**DOI:** 10.1038/s41420-025-02349-4

**Published:** 2025-02-20

**Authors:** Ying Sui, Ziyang Shen, Zhenling Wang, Jifeng Feng, Guoren Zhou

**Affiliations:** 1 The Affiliated Cancer Hospital of Nanjing Medical University, Jiangsu Cancer Hospital and Jiangsu Institute of Cancer Research, Nanjing, China; 2 Department of General Surgery, The First Affiliated Hospital of Nanjing Medical University, Nanjing, China

**Keywords:** Cancer metabolism, Insulin signalling

## Abstract

Recent progress in cancer metabolism research has identified lactylation as a critical post-translational modification influencing tumor development and progression. The process relies on lactate accumulation and the activation of lactate-sensitive acyltransferases. Beyond its role in epigenetic regulation, lactylation has emerged as a significant factor in tumor metabolism and evolution, offering fresh opportunities for developing targeted therapies that transcend traditional approaches. This review explores the growing importance of lactylation in cancer biology and highlights its potential for advancing diagnostic tools and therapeutic strategies.

## FACTS

- Lactylation, a novel post-translational modification utilizing lactate as a substrate, is crucial in regulating tumor metabolism, epigenetics, and the tumor immune microenvironment.
- The accumulation of lactate is essential for the activation of lactate-sensing acyltransferases and the occurrence of lactylation. This metabolic preference of tumors serves as the cradle for lactylation, giving new significance to the Warburg effect.
- Lactylation modifications span the entire course of tumor evolution, extending beyond regulating the tumor’s epigenetic modifications. They broadly affect tumor initiation, progression, and the remodeling of the tumor microenvironment.
- Building on the foundation of previous research on metabolism and targeted drugs, leveraging lactate sensors and lactylation regulatory mechanisms as potential targets for cancer treatment offers distinct advantages.

## Open questions

- **Q1 Acyltransferases Preference**
  For non-specific acyltransferases, is there a competitive relationship between acetyl-CoA and lactyl-CoA, and which acyltransferases prefer to bind?
- **Q2 Lactylation Erasers**
  If there are specific lactylation writers, are there specific lactylation erasers?
- **Q3 Order of Lactylation**
  In the tumor microenvironment, which occurs earlier: lactylation in immune cells or in the tumor cells themselves?
- **Q4 Role of Lactylation in Cancer**
  Does lactylation in tumors only have a promoting effect on cancer, is it a perfect target?

## Introduction

The Warburg effect, a hallmark of cancer and a key feature of tumor metabolic reprogramming describes the tendency of tumor cells to rely on glycolysis for energy production, even when sufficient oxygen is available [1, 2]. Lactate was once considered only a byproduct of glycolysis. However, it is now recognized as a versatile regulatory molecule within tumors. Beyond serving as an energy source for tumor growth, lactate plays a role in signal transduction, modulates the pH of the tumor microenvironment, and influences the metabolic behavior of immune cells [3, 4]. Recent advancements in proteomics have revealed lactylation, a novel regulatory mechanism in cancer that utilizes lactate as a substrate for acylation modifications. This discovery provides a new lens for exploring tumor biology, particularly in the context of lactate metabolism [5].

Gene expression is heavily influenced by chromatin remodeling, with dynamic, cell-specific regulatory modifications like histone acetylation and methylation playing a vital role. These reversible changes enable cells to adapt and regulate gene functions in response to environmental stress [6, 7]. Since its discovery, histone lysine lactylation (Kla) has emerged as a distinctive epigenetic modification due to its widespread occurrence, reliance on readily available glycolytic products, and dependence on substrate concentration [8–11]. Notably, lactylation is not confined to histones, highlighting its broad potential in tumors characterized by the Warburg effect [12, 13].

The interconnected roles of metabolism, epigenetics, and protein modification regulation have opened numerous pathways for cancer treatment [14–16]. In the early exploration of lactylation, understanding its relationship with metabolic reprogramming and tumor evolution is crucial. This knowledge lays the groundwork for advancing biological research and translating findings into clinical applications.

This review highlights the role of lactylation modifications in tumor development, progression, and the remodeling of the tumor immune microenvironment. It also discusses existing targets, drugs, and future research directions. By systematically categorizing the molecules involved in lactylation regulation and their associated therapeutic agents, this review aims to offer new insights into leveraging lactylation for early cancer diagnosis and targeted treatments.

## Results

### Lactate, lactylation, and tumorigenesis

Tumor development is intricately linked to the inactivation of tumor suppressor genes, with dysregulated cellular metabolism standing as a hallmark of cancer [1, 17]. The interaction between unchecked growth signals and tumorigenesis has been a focal point of research. In 2012, Contractor Tet et al. identified the pivotal role of P53 in regulating the Warburg effect. Their study proposed a novel mechanism in which P53 modulates PDK2 activity to shift tumor metabolic preferences, thereby promoting tumorigenesis [18]. Subsequently, classical oncogenes such as PTEN and KRAS were demonstrated to regulate lactate metabolism in tumor cells [19–21]. Lactate exhibits diverse pro-carcinogenic effects, including promoting early local tumor invasion by aiding extracellular matrix degradation and enhancing tumor angiogenesis. Additionally, elevated lactate concentrations in the tumor microenvironment influence the activity of T cells and macrophages, contributing to immune evasion during the early stages of tumor development [22–24]. Lactylation, a post-translational modification using lactate as a substrate, provides a key regulatory mechanism in early tumorigenesis. In tumor cells, AARS1 detects lactate and drives global lysine lactylation, including the lactylation of P53. This modification impairs the function of p53, suppressing its tumor-suppressive capabilities, and offers a novel perspective on the role of p53 dysfunction in cancer progression [17]. Glycolysis-driven lactylation of the nucleolar protein NCL by the enzyme P300 inhibits alternative splicing associated with the translation termination of downstream MADD. This process ultimately promotes tumor growth through the NCL/MADD/pERK axis, offering a new dimension to the activation of the MAPK signaling pathway in cancer progression [25]. The regulatory role of lactylation in tumorigenesis offers valuable insights and theoretical foundations for early cancer diagnosis. While current methods for detecting lactylation, such as metabolomics and antibody-based techniques, lack systematic clinical studies focused on diagnostics, the potential for leveraging this mechanism as a tool for early cancer detection is highly promising.

### Lactylation and tumor evolution

The excessive accumulation of lactate in tumors, driven by the Warburg effect or other metabolic shifts, supplies the substrate needed for intratumoral protein lactylation. This sets the stage for diverse molecule-mediated lactylation regulatory processes. Understanding this mechanism reveals a novel pathway for tumor evolution, extending beyond traditional DNA-level regulation.

#### Warburg effect and lactic acid-driven epigenetic control

The Warburg effect plays a crucial role in driving tumor cell growth, acidifying and reshaping the tumor microenvironment, and regulating tumor immunity [26]. This metabolic shift alters the availability of various intermediates that directly influence epigenetic modifications. For instance, acetyl-CoA, a key substrate for histone acetylation, experiences level fluctuations that impact global histone acetylation states [27]. Typically, cytoplasmic acetyl-CoA concentrations decrease, while mitochondrial levels remain stable or may slightly increase [28]. In the context of the Warburg effect, increased glycolysis reduces the amount of pyruvate entering the mitochondria. This diminishes the production of acetyl-CoA via the pyruvate dehydrogenase complex (PDC), potentially affecting histone acetylation and, consequently, gene expression [26, 27]. Additionally, lactate accumulation resulting from the Warburg effect significantly impacts the epigenetic regulation of tumor cells. This occurs through mechanisms such as cellular acidification, which affects the activity of histone deacetylases (HDACs), or alterations in the NAD+/NADH ratio, influencing the activity of the NAD+-dependent deacetylase SIRT1. These changes collectively modulate the global epigenetic landscape within tumor cells [29, 30]. Lactylation directly regulates downstream processes and functions as a rapid gene regulatory mechanism, comparable to acetylation. However, while some tumor cells may sustain acetyl-CoA levels through enhanced glutamine metabolism or fatty acid oxidation, lactylation often takes precedence in gene regulation due to its greater substrate availability [31]. Since Zhang et al. first identified lactylation in 2019, the field has advanced significantly, with H3K18La emerging as the most studied histone lactylation modification [32]. In neuroendocrine prostate cancer (NEPC), this modification facilitates cellular plasticity, playing a pivotal role in tumor evolution. The epithelial-mesenchymal transition (EMT) regulator ZEB1 influences H3K18La to enhance the expression of key glycolytic enzymes such as LDHA. This shift alters tumor metabolic preferences and increases chromatin accessibility near neuro-related genes, thereby promoting tumor cell plasticity and adaptability [33]. Research by Yu et al. revealed that H3K18La contributes to tumorigenesis by upregulating YTHDF2, an m6A reader, which promotes the degradation of PER1 and TP53 mRNA [34]. This discovery highlights a novel connection between the Warburg effect and epigenetic regulation, deepening our understanding of how metabolic and epigenetic pathways interact in cancer development.

#### Lactate sensors and lactylation control

**Fig. 1:**
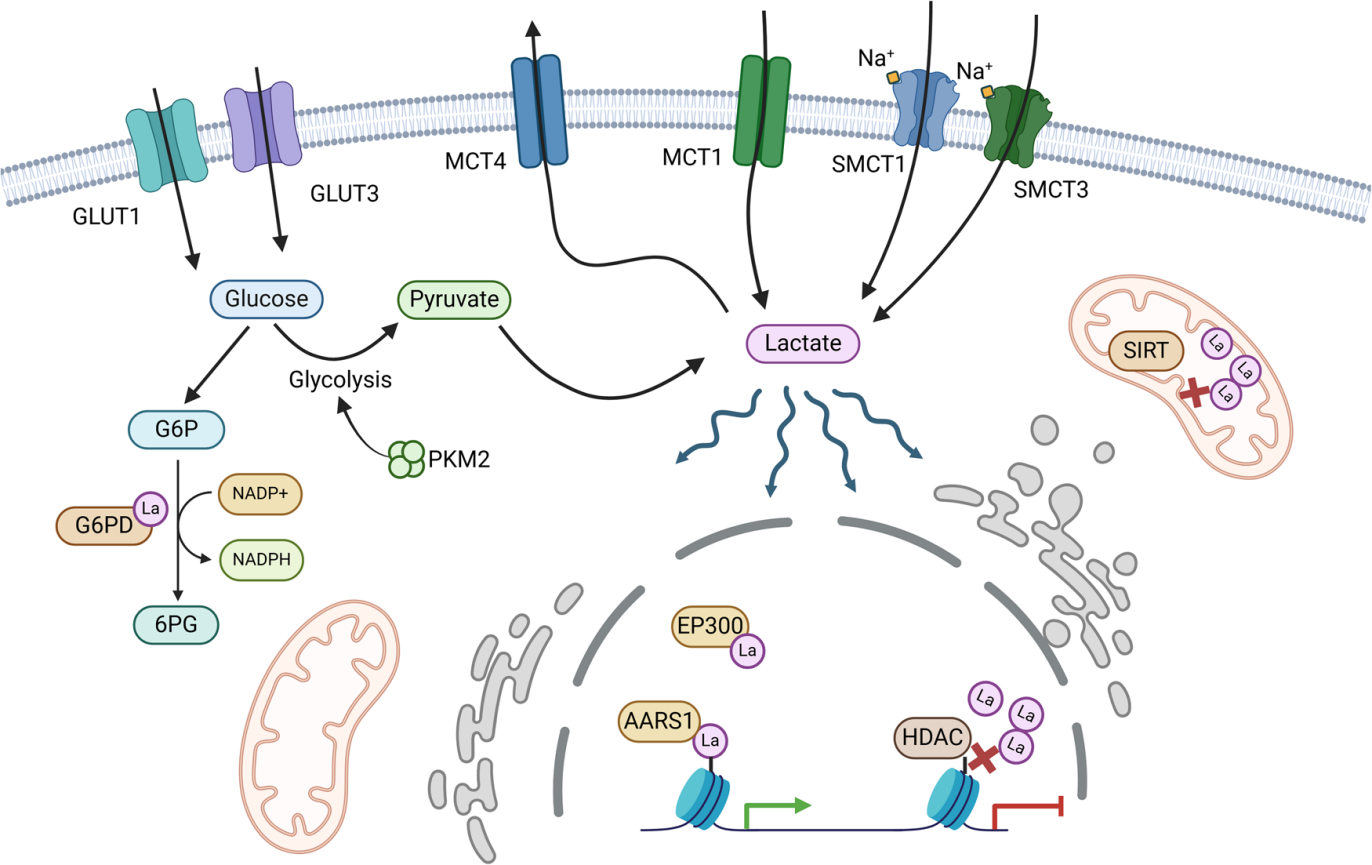
Reported and potential lactate sensors in cancer. Reported and potential lactylation-regulating genes,including lactylation-related metabolic transport enzymes, lactylation-related metabolic enzymes, lactyltransferases, and de-lactylases.

We have compiled a summary of potential targets and therapeutic agents aimed at controlling lactylation, presented in Table 1 and Fig. 1. Additionally, Table 2 provides detailed information on those targets and drugs currently in clinical trials, excluding any that have already reached commercialization. This overview offers a comprehensive perspective on the translational progress in this area of research.

**Table 1:** Lactylation regulator and drugs.

Targets	Inhibitor name	In clinical trial	Commercial drug	Reference
MCT1	AZD3965	Yes	–	[66]
	α-CHCA	No	–	[67]
MCT4	VB124	No	–	[68]
SMCT1	–	–	–	–
SMCT2	–	–	–	–
GLUT1	BAY-876	No	–	[69]
	WZB117	No	–	[70]
GLUT3	KL-11743	No	–	[71]
PKM2	PKM2-IN-1	No	–	[72]
G6PD	G6PDi-1	No	–	[73]
	RRx-001	Yes	–	[47]
EP300	SGC-CBP30	No	–	[74]
	PLX51107	No	–	[75]
AARS1	–	–	–	-
HDAC1	PCI-24781/CRA-024781	Yes	Abexinostat	[76]
	JNJ-26481585	Yes	Quisinostat	[77]
	MGCD0103/MG0103	Yes	Mocetinostat	[78]
	Pyroxamide	No	–	[79]
HDAC1/HDAC2	FK228/FR901228	Yes	Romidepsin	[77]
HDAC2	Santacruzamate A	No	–	[80]
HDAC1/HDAC3	MS-275/SNDX-275	Yes	Entinostat	[81]
HDAC3	RGFP966	No	–	[82]
SIRT2	AGK2	No	–	[83]
	Thiomyristoyl	No	–	[84]
SIRT3	3-TYP	No	–	[85]

Molecules associated with lactylation regulation and targeted therapies against them. Drugs that have been commercialized or are under clinical trials are listed separately.

**Table 2:** Lactylation target drugs in clinical trials.

Drug/Target	Clinicaltrials.gov identifier	Cancer	Type of Trial	Population (N, age)	Intervention	Objective	Primary outcome/endpoint	Status
AZD3965/MCT1	NCT01791595	Advanced solid tumors or lymphomas	Phase 1	53, age ≥ 18	Capsule taken daily	Find out the maximum dose that can be given safely to patients	MTD of AZD3965	Completed (2013-04-23~2020-11-17)
RRx-001/G6PD	NCT01359982	Advanced solid tumors or lymphomas	Phase 1	26, age ≥ 18	Intravenous infusions	Evaluate the safety and pharmacokinetic profile of RRx-001	Number, frequency, and type of adverse events	Completed (2011-09~2015-02)
	NCT02096341	Advanced Solid Tumors or lymphoma	Phase 1	2, age ≥ 18	Subcutaneous injections	Investigate the dosage of RRx-001 by the subcutaneous route	Number of Participants with Serious and Non-Serious Adverse Events	Terminated (2014-04~2016-01)
	NCT02096354	Advanced or metastatic colorectal cancer	Phase 2	62, age ≥ 18	Intravenous infusions	Compare the safety and activity between RRx-001 against regorafenib followed by irinotecan-based therapies	Overall Survival(OS)	Completed (2014-05-~2018-04-13)
	NCT02215512	Participants with brain metastases	Phase 1	29, age ≥ 18	Intravenous infusions	test the safety and activity of whole brain radiation therapy with RRx-001	Number, frequency, and type of adverse events	Completed (2015-02-06-~2016-11-28
	NCT02489903	Platinum Refractory/Resistant Small Cell Carcinoma, EGFR TKI Resistant EGFR + T790M Negative Non-Small Cell Lung Cancer, High-Grade Neuroendocrine Tumors and Resistant/Refractory Ovarian Cancer	Phase 2	139, age ≥ 18	Intravenous infusions	Explore the potential of RRx-001 to sensitize patients who previously received and now have failed a platinum-based doublet regimen	OS	Completed (2015-06-~2021-12-06)
	NCT02518958	Advanced, malignant, solid tumor(s) or lymphoma	Phase 1	12, age ≥ 18	Intravenous infusions	Determine the feasibility of co-administration of RRx-001 and nivolumab	Number, frequency, and type of adverse events	Completed (2015-07-21~2016-05-17)
	NCT02452970	Biliary tract adenocarcinoma/cholangiocarcinoma	Phase 2	4, age ≥ 18	Intravenous infusions	See if RRx-001 can resensitize the tumor(s)	Overall Objective Response (RECIST)	Terminated (2015-07-16~2016-05-10)
	NCT02801097	Advanced, malignant, solid tumor(s)	Phase 1	28, age ≥ 18	Intravenous infusion	Evaluate the safety, pharmacodynamics, and clinical activity of RRx-001 administered in combination with irinotecan	Number, frequency, and type of adverse events	Terminated (2016-08-30~2018-11-22)
	NCT02871843	High-grade glioma	Phase 1	19, age ≥ 18	Intravenous infusion	Tests The safety, tolerability, and activity of RRx-001, with standard radiation and temozolomide followed by temozolomide + RRx-001 in newly diagnosed GBM.	Number, frequency, and type of adverse events	Completed (2017-02-14~2019-10-11)
	NCT03515538	SCC of the oral cavity and oropharynx	Phase 2	48, age≥18	Intravenous infusion	Determine if RRx-001 reduces the duration or length of severe oral mucositis in patients with head and neck cancers	Duration of Severe Oral Mucositis (SOM)	Completed (2018-07-12~2019-10-22)
	NCT04525014	Recurrent or progressive malignant (World Health Organization (WHO) grade 3 or 4 tumors) primary brain or spinal cord tumors and solid tumors (excluding lymphomas)	Phase 1	2, age≥18	Intravenous infusion	Tests the experimental drug RRx-001 in combination with irinotecan and temozolomide in patients with cancer.	Recommended phase 2 dose	Terminated (2023-01-26~2024-07-29)
	NCT05566041	Small cell cancer (SCC)	Phase 3	292, age ≥ 18	Intravenous infusion	find out whether RRx-001 + platinum chemotherapy is more effective than platinum chemotherapy alone in 3rd line or beyond small cell cancer	Progression Free Survival (PFS) and OS	Recruiting (2022-08-01~2025-12-31)
	NCT05966194	SCC of the oral cavity or oropharynx	Phase 2	216, age ≥ 18	Intravenous infusion	Determine if RRx-001 reduces the incidence of severe oral mucositis in patients with head and neck cancers	Incidence of SOM through Intensity-modulated radiation therapy (IMRT)	Recruiting (2024-04-02~2025-07-01)

Detailed information on clinical trials of targeted lactylation drugs, including objectives, population, trial status, and primary endpoints.

##### Lactylation-related metabolic transport enzymes

Members of the monocarboxylate transporter (MCT) protein family are known not only for their traditional role in transporting monocarboxylates such as lactate across membranes but also for their involvement in lactate sensing and lactylation regulation. MCT1, primarily responsible for lactate uptake, is abundant in oxidative tumor cells, which utilize lactate from the tumor microenvironment as an energy source [35]. MCT1-mediated lactylation plays a key role in stabilizing HIF1A, enhancing KIAA1199 transcription to promote tumor angiogenesis [36]. Additionally, it influences tumor-infiltrating macrophages by driving their polarization toward the M2 phenotype through increased TNFSF9 expression via H3K18La [37]. MCT4 has also been implicated in the regulation of macrophage polarization from M1 to M2 phenotypes via H3K8la in arteriosclerosis, though similar mechanisms in tumors remain unexplored [38]. The MCT1 inhibitor AZD3965 has completed phase I clinical trials for advanced solid tumors and lymphomas, demonstrating tolerability at doses sufficient to achieve target engagement, and offering promise as a therapeutic agent [39].

In addition to MCT family members, sodium-coupled monocarboxylate transporters (SMCTs), such as SMCT1 and SMCT2, also play a role in cellular metabolism by co-transporting lactate along with sodium ions, utilizing the sodium ion concentration gradient [40, 41]. This mechanism parallels that of the sodium-glucose co-transporter (SGLT2), which is crucial in renal glucose reabsorption [42]. The potential connection between SMCTs and lactylation regulation opens an intriguing avenue for future research, providing further insight into the interplay between cellular transport systems and epigenetic modifications. This linkage could advance our understanding of tumor metabolism and therapeutic targets.

Beyond monocarboxylate transporters, glucose transporters have also been implicated in regulating lactylation. GLUT3 was identified as a regulator of protein lactylation in gastric cancer, marking the first report of a glucose transporter influencing intracellular lactylation in oncology [43]. Further research by Alessandra De Leo et al. demonstrated in glioblastomas that GLUT1 expression in glycolytic monocyte-derived macrophages (MDMs) facilitated intracellular lactate-driven histone lactylation near the IL-10 gene. This process led to increased IL-10 accumulation in the tumor microenvironment, suppressing T-cell activity [44]. These findings provide a novel perspective on the traditional roles of glycolysis-related transporters, highlighting their contributions to tumor metabolism and immune modulation.

##### Lactylation-related metabolic enzymes

Key glycolysis-related enzymes have long been pivotal in studying tumor metabolism and exploring translational applications. Protein lactylation, driven by lactate accumulation, introduces a novel mechanism for regulating tumor functions through metabolic enzymes.

PKM2, a pyruvate kinase isozyme, is highly expressed in rapidly proliferating cancer cells, catalyzing the final step of glycolysis to produce pyruvate and ATP. PKM2 alternates between an active tetrameric form and a less active dimeric form [45]. In glioblastomas, the interaction of ALDH1A3 with PKM2 promotes tetramer formation, leading to lactate accumulation in cancer stem cells. This, in turn, triggers lactylation-induced nuclear translocation of XRCC1, enhancing DNA repair and potentially contributing to tumor resistance to radiotherapy and chemotherapy [13]. Lactylation also impacts non-glycolytic metabolism. Tumor cells, often exposed to oxidative stress, rely on G6PD to generate NADPH, which is crucial for managing oxidative stress and supporting proliferation through the pentose phosphate pathway (PPP). In HPV-related cervical cancer, the HPV16 E6E7 protein inhibits lactylation of G6PD at the K45 site, promoting G6PD dimerization and enhancing its activity. This activation of the PPP fuels rapid tumor growth [46]. RRx-001, a G6PD inhibitor, has been under clinical investigation since 2011 for advanced malignancies, including colorectal cancer, lymphoma, and head and neck cancer, with promising outcomes [47]. Its latest phase III clinical trial targets small-cell lung cancer and is currently recruiting, offering potential advancements in cancer therapy.

##### Lactyltransferases

P300 (EP300) was initially identified as a transcriptional coactivator that enhances gene expression by interacting with various transcription factors. In 2019, Zhang et al. proposed its potential role as a lactyltransferase [32]. Subsequent studies confirmed P300’s lactyltransferase activity in cancers such as pancreatic ductal adenocarcinoma (PDAC) and intrahepatic cholangiocarcinoma, implicating it in lactylation regulation [31, 48]. However, P300 is not exclusive to lactylation—it also catalyzes other acyl modifications. P300 serves as a critical integrator of metabolic and transcriptional signals. By regulating acetylation with interactions involving MYC, AKT, and TGF-β, P300 drives epigenetic changes that promote tumor growth and progression [49–51]. The proportion or preference of P300-mediated protein lactylation and acetylation, as well as their impact on regulatory networks, await further research and reporting. Understanding this crosstalk could open new avenues for targeted cancer therapies. AARS1 was the first identified lactyltransferase and plays a critical role in sensing intracellular lactate. It translocates to the nucleus, where it lactylates and activates the YAP-TEAD complex, a key component of the Hippo pathway. This process is subject to positive feedback regulation by YAP-TEAD itself [12]. Similarly, the acetyltransferase KAT5 (TIP60), known for facilitating histone acetylation, also mediates lactylation. KAT5/TIP60-driven lactylation of PIK3C3/VPS34 at lysine residues 356 and 781 enhances its interaction with BECN1, ATG14, and UVRAG, promoting autophagy that may contribute to cancer progression [52]. Research on lactylation-specific “writers” remains in its infancy, and further studies are needed to elucidate their precise roles and mechanisms in cancer biology.

##### De-lactylases

The HDAC (histone deacetylase) family was originally identified for its role in regulating chromatin structure and gene expression by removing acetyl groups from histones [53]. Building on their discovery of P300 as a lactyl “writer,” Professor Zhao Yingming’s team reported that common deacetylases, including HDAC1-3 and SIRT1-3, can remove lactylation modifications, identifying them as potential lactyl “erasers.” They further proposed the specificity of lactylation sites and the possibility of other lactyl group erasers yet to be discovered [54]. Class I HDACs (HDAC1-3) were confirmed by Zhao’s team as the most effective lysine de-lactylases in vitro. Despite this, reports linking HDACs to the regulation of lactylation in cancer are scarce. Notably, HDAC inhibitors, such as tucidinostat, are already approved for cancer treatment, suggesting potential for therapeutic exploration in targeting lactylation pathways via HDAC modulation. Further research is needed to better understand the role of HDACs in lactylation regulation and their implications in cancer therapy [55].

Vorinostat (SAHA, Zolinza) was the first HDAC inhibitor approved by the FDA in 2006, achieving a total remission rate of 30% in patients with recurrent or refractory cutaneous T-cell lymphoma (CTCL) [56]. Since then, HDAC inhibitors have undergone significant advancements, demonstrating efficacy in select conditions. However, their broader application, particularly as monotherapies for solid tumors, has remained limited [57]. Research into HDAC regulation of lactylation may pave the way for a new generation of HDAC inhibitors with enhanced specificity and efficacy.

SIRTs (NAD+-dependent deacetylases) also play a role in tumor suppression. SIRT2 and SIRT3 have been reported to inhibit tumor progression by de-lactylating non-histone proteins [58, 59]. Despite the absence of lactyl group-specific de-lactylases, the growing body of research on deacetylases suggests significant potential for identifying “erasers” of lactylation modifications.

Further investigation into these acyl modification “erasers” could deepen our understanding of their regulatory mechanisms in cancer and inspire new strategies for clinical applications. By bridging insights from lactylation biology and therapeutic development, these studies hold promise for more targeted and effective cancer treatments.

### Lactylation and tumor microenvironment remodeling

Lactylation utilizes the metabolic products of glucose, namely lactate, and ATP, to covalently modify proteins, achieving regulatory effects. This mechanism shares similarities with ATP-dependent phosphorylation, suggesting that lactylation may serve as a convenient and widespread regulatory method. Over time, numerous key proteins and prominent cell types have been identified as participants in this novel process.

**Fig. 2:**
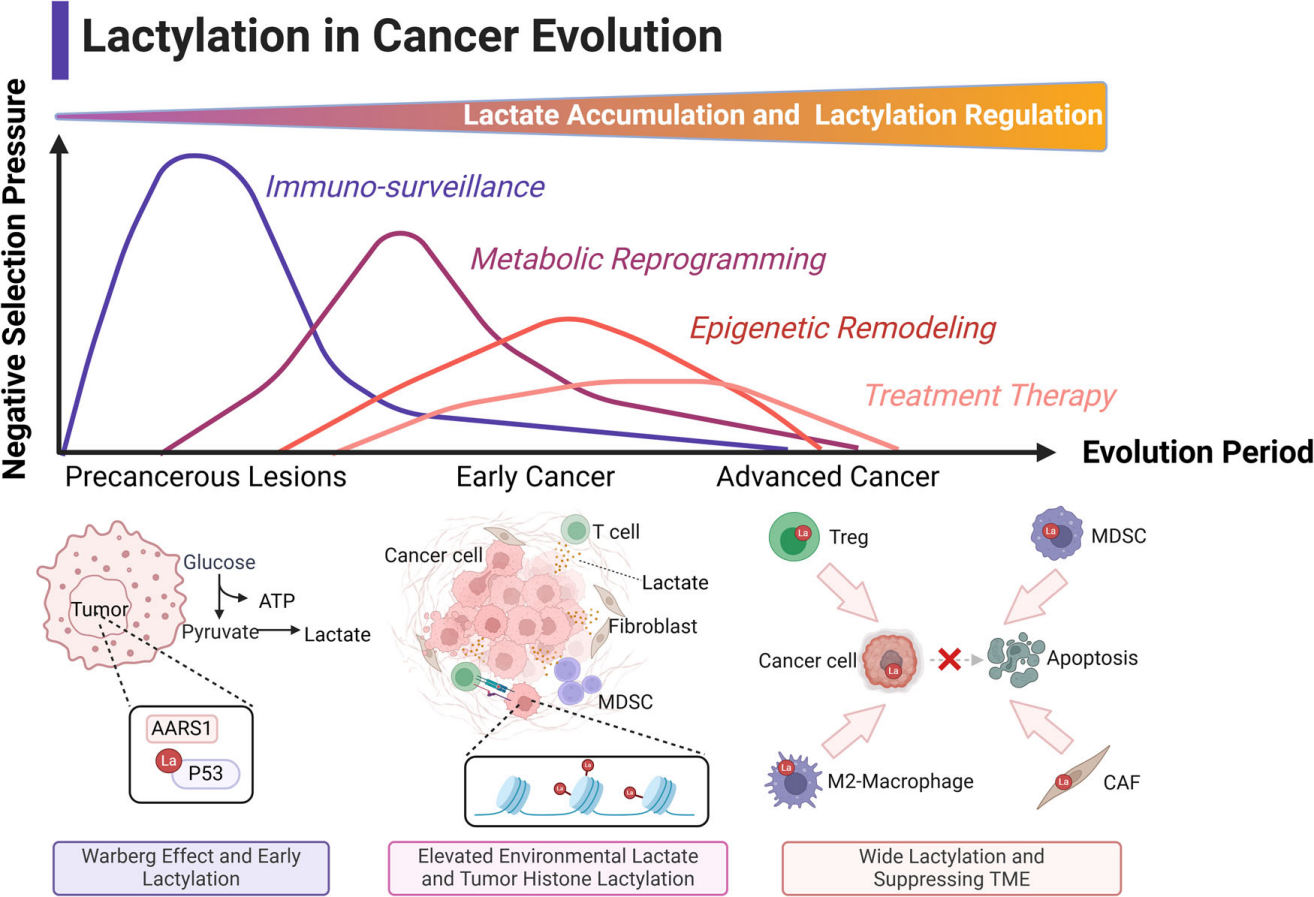
Lactylation in cancer evolution. The occurrence of lactylation spans the entire process of tumor initiation and progression, impacting the tumor immune microenvironment, epigenetic regulation, metabolism, and therapeutic outcomes.

Current research predominantly focuses on the role of lactylation in mediating epigenetic changes in immune cells under lactate stress within the tumor microenvironment. However, this raises intriguing questions about whether other critical intracellular targets are influenced by lactate and operate through lactylation. Exploring these mechanisms further could uncover additional pathways and targets affected by this regulatory mode, expanding our understanding of lactylation’s role in cellular and tumor biology (Fig. 2).

#### Lactylation in immune cells: epigenetic control

The Warburg effect in tumors drives lactate accumulation in the tumor microenvironment, reshaping immune cell metabolism and perpetuating a cycle of malignant tumor behavior, immune cell remodeling, and environmental immunosuppression [3]. Tumor-intrinsic PI3K pathways amplify lactate production and accumulation, exacerbating this cycle. Under metabolic stress, tumor-associated macrophages (TAMs) exhibit increased histone lactylation at H3K18, reducing their anti-cancer phagocytic activity [21]. Jia Xiong et al. found that lactate accumulation induces upregulation of METTL3 in tumor-infiltrating myeloid cells (TIMs) via H3K18la. This upregulation promotes m6A modification of Jak1 mRNA, enhancing protein translation and activating the JAK-STAT pathway, which further supports the immunosuppressive role of TIMs [60]. Similarly, within the STAT family, STAT5 has been shown to elevate PD-L1 expression on leukemia cells through histone lactylation, suppressing normal T-cell activation and contributing to immune evasion. These findings underscore the critical role of lactylation in epigenetically modulating immune responses within the tumor microenvironment [61].

#### Immunosuppressive signaling enhancement

Lactylation at key signaling sites within the tumor microenvironment amplifies immunosuppressive signaling, undermining normal tumor immunity. For instance, lactate-driven lactylation of MOESIN enhances the production of regulatory T cells (Tregs) in the tumor microenvironment. This modification improves MOESIN’s interaction with the TGF-β receptor, activating the classical SMAD3 signaling pathway and fostering immune suppression [62]. In innate immunity, the cGAS protein is essential for immune surveillance, detecting mitochondrial DNA (mtDNA) or chromosomal DNA fragments. However, lactylation of cGAS by AARS2 inactivates cGAS, suppressing the synthesis of cGAMP and impairing the innate immune response. Studies show that blocking monocarboxylate transporter 1 (MCT1) to inhibit L-lactate transport can prevent cGAS lactylation, thereby restoring innate immunity [63]. While cGAS lactylation has not yet been reported in oncology, these findings expand our understanding of the interplay between classic immune pathways and lactylation. This knowledge offers valuable insights and a theoretical framework for developing innovative immunotherapeutic strategies targeting lactylation-driven immune dysregulation.

## Concluding remarks

Lactylation, a novel post-translational modification (PTM) utilizing lactyl residues, has significantly advanced our understanding of lactate’s role in tumor initiation and progression. This modification not only impacts histones by altering chromatin structure and DNA accessibility to regulate gene expression but also broadly influences the activity of key molecules, akin to phosphorylation. The close relationship between lactate concentration and lactylation establishes a direct link between metabolic dysregulation in tumors and other cancer hallmarks, such as sustained proliferation signals and immune evasion [1, 64].

Research on lactylation in tumors builds upon foundational discoveries in tumor metabolism, paving the way for clinical translation based on metabolic and lactylation regulatory mechanisms. While concerns persist about the specificity of lactylation-related “writers” and “erasers” posing challenges for targeted therapies, the identification of AARS1/2 as specific lactylation writers offer hope for precise regulation and therapeutic innovation.

The translational challenges are considerable, as many inhibitors and drugs focus on enzymes like LDH, MCTs, and PKM. These enzymes, similar to lactylation “writers” and “erasers,” also regulate other critical metabolic reactions, necessitating careful evaluation to balance potential benefits and risks in tumor patients. For instance, while alanine has been identified as a competitive inhibitor of AARS1, gaps in our understanding of its broader implications remain significant [65].

Although lactate accumulation is a necessary condition and environmental feature for lactylation, the potential influence and regulation of other specific tumor microenvironments, such as hypoxia and inflammatory conditions, cannot be excluded. This part of the puzzle urgently awaits further research for clarification. Moreover, different cancer types exhibit varying metabolic preferences. Tumors originating from tissues highly reliant on glycolytic metabolism or those from tissues that minimally utilize glycolysis for energy may possess distinct lactylation characteristics. Most studies suggest that histone lactylation supports tumor progression, often promoting tumor growth. However, whether lactylation is inherently pro-carcinogenic or context-dependent requires further exploration. Current research has only touched the surface of lactylation’s potential as a target in cancer therapy. It is not merely an epigenetic pathway and should not be approached simplistically as such.

Further investigation into lactylation’s role in tumor initiation and the regulation of the tumor immune microenvironment holds promise for breakthroughs in cancer diagnostics and treatment. In particular, lactylation detection could enable early diagnosis, and immunomodulatory drugs that inhibit lactylation may emerge as effective therapeutic strategies. These advancements could redefine the landscape of cancer care by integrating metabolic and immunological perspectives.
